# SREBP2 as a central player in cancer progression: potential for targeted therapeutics

**DOI:** 10.3389/fphar.2025.1535691

**Published:** 2025-04-16

**Authors:** Ruiqi Chen, Tianyu Chen, Xiang Li, Junfeng Yu, Min Lin, Siqi Wen, Man Zhang, Jinchi Chen, Bei Yi, Huage Zhong, Zhao Li

**Affiliations:** ^1^ Division of Colorectal and Anal Surgery, Department of Gastrointestinal Surgery, Guangxi Medical University Cancer Hospital, Nanning, China; ^2^ Department of Experimental Research, Guangxi Medical University Cancer Hospital, Nanning, China; ^3^ Guangxi Clinical Research Center for Colorectal Cancer, Nanning, China

**Keywords:** SREBP2, cholesterol metabolism, cancer, tumor microenvironment, cancer therapy

## Abstract

Recent studies have identified the reprogramming of lipid metabolism as a critical hallmark of malignancy. Enhanced cholesterol uptake and increased cholesterol biosynthesis significantly contribute to the rapid growth of tumors, with cholesterol also playing essential roles in cellular signaling pathways. Targeting cholesterol metabolism has emerged as a promising therapeutic strategy in oncology. The sterol regulatory element-binding protein-2 (SREBP2) serves as a primary transcriptional regulator of genes involved in cholesterol biosynthesis and is crucial for maintaining cholesterol homeostasis. Numerous studies have reported the upregulation of SREBP2 across various cancers, facilitating tumor progression. This review aims to provide a comprehensive overview of the structure, biological functions, and regulatory mechanisms of SREBP2. Furthermore, we summarize that SREBP2 plays a crucial role in various cancers and tumor microenvironment primarily by regulating cholesterol, as well as through several non-cholesterol pathways. We also particularly emphasize therapeutic agents targeting SREBP2 that are currently under investigation. This review seeks to enhance our understanding of SREBP2’s involvement in cancer and provide theoretical references for cancer therapies that target SREBP2.

## 1 Introduction

Lipids comprise a diverse array of molecules that serve as essential components of biological membranes and are widely distributed across cellular organelles (Pomorski et al., 2001; Efeyan et al., 2015). Cholesterol is a lipid that primarily regulates the rigidity, fluidity, and permeability of the lipid bilayer in cell membranes, and also plays a critical role in signal transduction, promoting diverse cellular functions (Goldstein et al., 2006; Menendez and Lupu, 2007). Dysregulation of cholesterol metabolism can initiate or exacerbate the progression of numerous diseases (Cohen et al., 2011; Schwartz et al., 2013; Goldstein and Brown, 2015). Recent reports indicate a significant upregulation of cholesterol biosynthesis in human cancers, as elevated synthesis and uptake of cholesterol are necessary to meet the demands of membrane biogenesis and support ongoing cellular replication (Gruenbacher and Thurnher, 2015; Bathaie et al., 2017). In the tumor microenvironment, cancer cells exploit cholesterol metabolism to facilitate rapid migration, invasion, and metastasis (Bian et al., 2021).

Lipid metabolism is transcriptionally regulated by sterol regulatory element-binding proteins (SREBPs) (Horton et al., 2002). First isolated from the nuclei of HeLa cells in 1993, SREBPs belong to the basic helix-loop-helix–leucine zipper (bHLH-Zip) family of transcription factors (Briggs et al., 1993; Wang et al., 1993; Brown and Goldstein, 1997). The SREBPs family comprises three subtypes, SREBP1a and SREBP1c, SREBP2(Brown and Goldstein, 1997). SREBP2 predominantly activates the transcription of key genes in the mevalonate (MVA) pathway, thereby regulating cholesterol synthesis (Hua et al., 1993; Horton et al., 2002; Horton et al., 2003). SREBP2-mediated cholesterol metabolism plays a crucial role in various cancers, including lung cancer, colorectal cancer, and breast cancer, among others. In this review, we first introduce the structure, biological processes, and recent advances in the regulation of SREBP2. Subsequently, we summarize the role of SREBP2 in different types of cancer and tumor microenvironment. Given the emergence of SREBP2 as a significant target for cancer therapy, we focus on and discuss several important SREBP2-targeting drugs. Through these discussions, this article aims to provide new insights into potential cancer therapies.

## 2 SREBP2 structure

**FIGURE 1:**
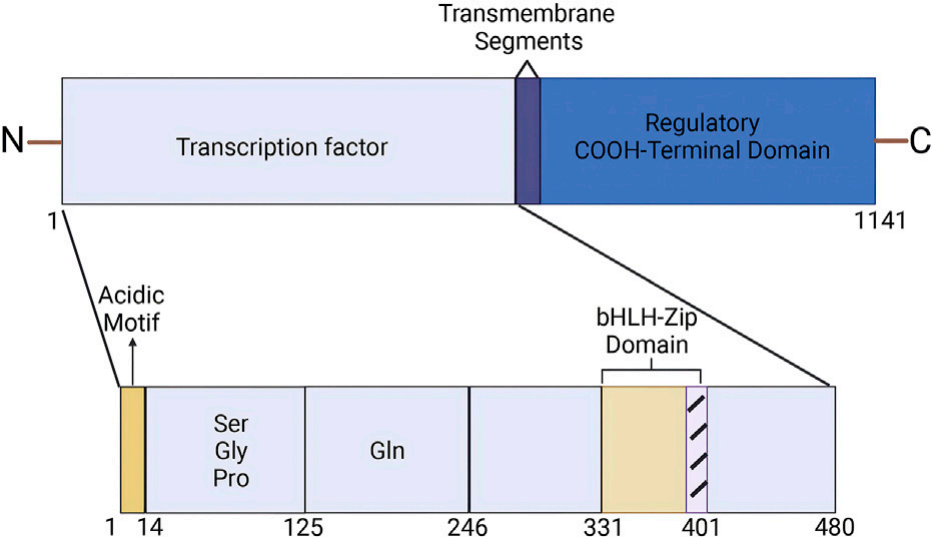
Structure of SREBP2. (Created in BioRender. Chen, R. (2025) https://BioRender.com/r73a111). The SREBP2 protein comprises three main segments: an NH2-terminal domain of approximately 480 amino acids, a middle hydrophobic region of approximately 80 amino acids, and a COOH-terminal domain of approximately 590 amino acids. The NH2-terminal domain contains an acidic region that is responsible for transcriptional activation, as well as a basic helix-loop-helix-leucine zipper (bHLH-Zip) motif that specifically binds to DNA sequences.

The human SREBP2 gene is located on chromosome 22q13 and encodes a protein consisting of 1,141 amino acids. It was first cloned and characterized by Xianxin Hua et al., in 1993, who isolated cDNA from cultured HeLa cells (Hua et al., 1993; Miserez et al., 1997). The SREBP2 protein comprises three main segments: (a) an NH2-terminal domain of approximately 480 amino acids, which contains an acidic region responsible for transcriptional activation, along with a basic helix-loop-helix-leucine zipper (bHLH-Zip) motif that specifically binds to DNA sequences; (b) a middle hydrophobic region of approximately 80 amino acids, which includes two hydrophobic transmembrane segments; and (c) a COOH-terminal domain of approximately 590 amino acids (Brown and Goldstein, 1997) (Figure 1).

The hydrolyzed activated NH2-terminal of SREBP2 binds to sterol regulatory element (SRE) sequences in the promoters of target genes in the nucleus, thereby upregulating their transcription (Goldstein et al., 2006). The middle hydrophobic region features two hydrophobic membrane-spanning sequences and a hydrophilic lumenal loop that separates these segments (Luo et al., 2020). This hydrophilic luminal loop extends into the endoplasmic reticulum (ER) lumen (Weber et al., 2004; Luo et al., 2020). The COOH-terminal domain of SREBP2, referred to as the regulatory domain, interacts with the WD-repeat domain of SREBP-cleavage activating protein (SCAP) (Gong et al., 2016; Brown et al., 2018).

## 3 Regulation of SREBP2

The SREBP2 gene is transcribed and translated into SREBP2 precursor (Pre -SREBP2), which is then anchored in the endoplasmic reticulum. To become active, Pre -SREBP2 must exit the ER and undergo cleavage in the Golgi apparatus. This cleavage releases the N-terminal domain (n-SREBP2), which then translocates to the nucleus to activate the transcription of downstream target genes. This process is regulated by various mechanisms, as discussed below.

### 3.1 Transcription regulation of the SREBP2

**FIGURE 2:**
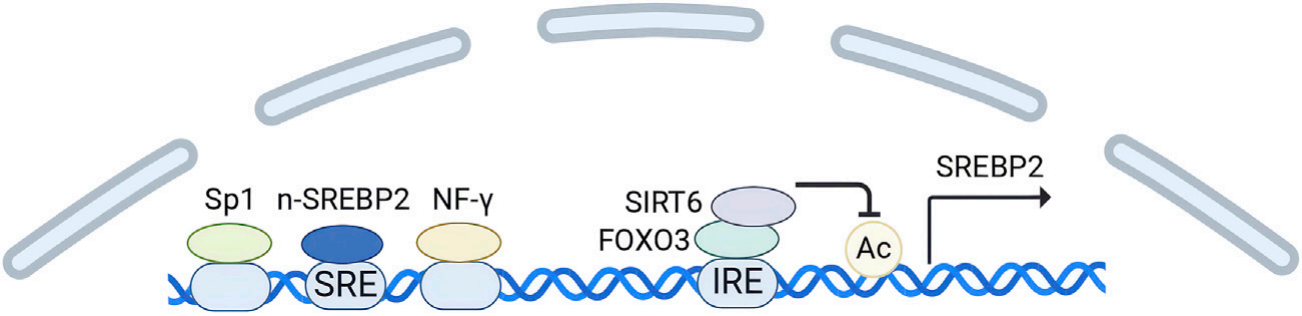
Transcription regulation of SREBP2. (Created in BioRender. Chen, R. (2024) https://BioRender.com/h24n110). The transcription start site of SREBP2 contains binding sites for the transcription factors SP1 and NF-Y, along with a 10-base pair SRE. SP1 and NF-Y cooperate with n-SREBP2 to upregulate the transcription of SREBP2. FOXO3 acts as a negative regulator by binding to a conserved insulin response element (IRE) in the SREBP2 gene and recruiting SIRT6. Subsequently, SIRT6 deacetylates histone H3 in the SREBP2 gene promoter, thereby suppressing SREBP2 expression.

Notably, because there is a 10-base pair SRE upstream of the transcription start site of SREBP2, it is also regulated by n-SREBP2(Sato et al., 1996). In addition, the region upstream contains binding sites for the transcription factors SP1 and NF-Y (Sato et al., 1996). Both of them cooperate with n-SREBP2 to upregulate the transcription of SREBP2. The key negative regulators of SREBP2 gene expression include SIRT6 and forkhead box O (FOXO3) (Tao et al., 2013). FOXO3 binds to a conserved insulin response element (IRE) in the SREBP2 gene and recruits SIRT6(Tao et al., 2013). Subsequently, SIRT6 deacetylates histone H3 at lysine residues 9 and 56 on the SREBP2 gene promoter (Tao et al., 2013). This modification promotes a repressive chromatin state, thereby inhibiting the expression of SREBP2 and its target genes (Figure 2).

### 3.2 Post-transcriptional regulation of the SREBP2

**FIGURE 3:**
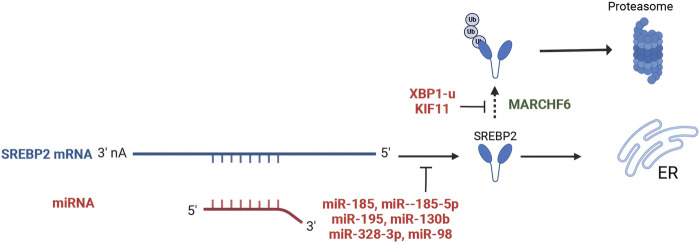
Post-transcriptional regulation and post-translational modifications of SREBP2. (Created in BioRender. Chen, R. (2024) https://BioRender.com/q25n513). miR-185, miR-185-5p, miR-195, miR-130b, miR-328-3p, and miR-98 directly bind to the 3′-untranslated region (3′UTR) of SREBP2 mRNA, leading to the inhibition of its expression. MARCHF6 serves as a primary ubiquitin ligase that promotes SREBP2 degradation. XBP1-u and KIF11 inhibit the ubiquitination and proteasomal degradation of SREBP2.

SREBP2 is post-transcriptionally regulated by microRNAs (miRNAs), which are small endogenous non-coding RNAs that exert their effects by binding to target mRNAs (Bartel, 2004). Studies demonstrated that miR-185 significantly reduced the levels of both full-length and mature SREBP2 proteins in liver cancer and prostate cancer cells by binding to the 3′untranslated region (3′UTR) of SREBP2 mRNA (Li et al., 2013; Yang et al., 2014; Chen et al., 2021). *In vivo* experiments also revealed that the miR-185 sequences resulted in decreased SREBP2 levels (Yang et al., 2014). However, inhibiting miR-185 did not lead to increased mRNA levels of SREBP2 or its downstream targets *in vivo* and vitro, suggesting that basal miR-185 may not significantly repress SREBP2 in these contexts (Chen et al., 2021). Additionally, several oncological studies have demonstrated that miR-185-5p, miR-195, miR-130b, miR-328-3p, and miR-98 directly target SREBP2 gene, leading to the inhibition of its expression (Geng et al., 2018; Yu et al., 2019; Huang et al., 2022; Tan et al., 2022; Tang et al., 2023). Overexpression of miR-27a and miR-10a was also found to decrease SREBP2 levels, although the mechanisms underlying these effects remain to be elucidated (Shirasaki et al., 2013; Horii et al., 2019) (Figure 3).

### 3.3 Post-translational modification of the SREBP2

Ubiquitination of the SREBP2 protein is a key post-translational modification. MARCHF6 serves as a primary ubiquitin ligase SREBP2 degradation (Dickson et al., 2023). However, XBP1-u inhibits the ubiquitination and proteasomal degradation of SREBP2, then stabilizing the protein in HCC(Wei et al., 2022). In pancreatic cancer, kinesin family member 11 (KIF11) interacts with SREBP2, increasing its protein levels by attenuating ubiquitination-mediated degradation (Gu et al., 2022) (Figure 3). The degradation of SREBP2 is regulated by its binding to SCAP. Early studies demonstrated that SREBP2 precursor levels are markedly reduced in the absence of SCAP (Rawson et al., 1999; Shao and Espenshade, 2014). Recent investigations have identified two distinct motifs within the carboxyl-terminal domain (CTD) of SREBP2: one functioning as a protective signal and the other as a degradation signal. When SREBP2 dissociates from SCAP, the degradation signal triggers its proteasomal degradation in the endoplasmic reticulum. Conversely, the protective signal motif enables SREBP2 to bind SCAP, thereby masking the degradation signal and stabilizing the protein (Kober et al., 2020).

### 3.4 Regulation of SREBP2 protein egress from the ER

**FIGURE 4:**
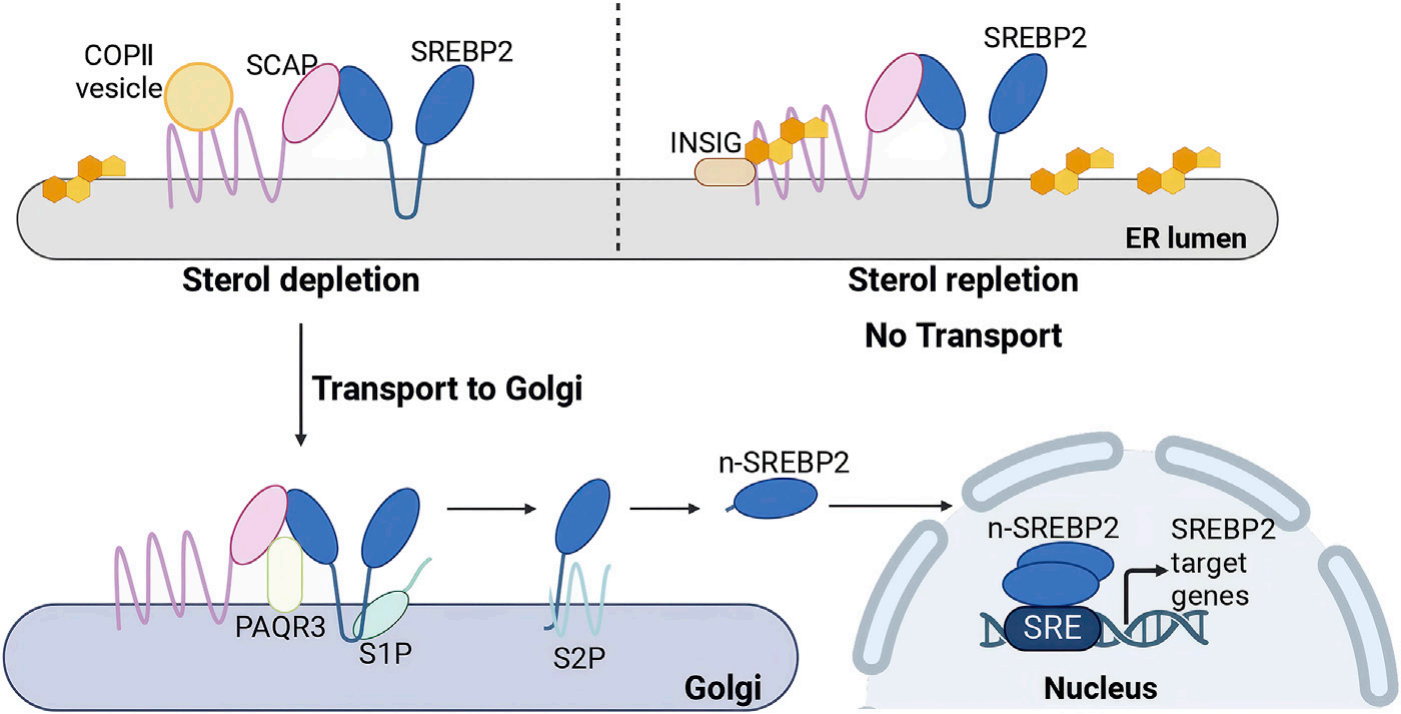
Activation regulation of SREBP2. (Created in BioRender. Chen, R. (2025) https://BioRender.com/xugbutl). Under cholesterol depletion: The SCAP-SREBP2 complex binds to COPII vesicles, facilitating its translocation to the Golgi apparatus. PAQR3 interacts with the SCAP-SREBP2 complex in the Golgi, retaining it within this organelle. Sequential proteolytic cleavage by Site-1 protease (S1P) and Site-2 protease (S2P) generates nuclear SREBP2 (n-SREBP2), which translocates to the nucleus as a homodimer to bind sterol regulatory elements (SREs) and activate transcription of target genes. Under cholesterol repletion: SCAP binds to INSIG, forming an ER-anchored INSIG/SCAP/SREBP complex that inhibits the transport of SREBP2 to the Golgi apparatus.

The SREBP2 precursor binds to sterol regulatory element-binding protein-cleavage activating protein (SCAP) via its C-terminal domain, anchoring it within the ER. The transfer of the SREBP2-SCAP complex is regulated by cholesterol levels. SCAP undergoes conformational changes in response to fluctuations in ER cholesterol levels (Brown et al., 2018). Recent cryoelectron microscopy (cryo-EM) structures suggest that Scap’s two ER luminal loops (loop 1 and loop 7), which flank an intramembrane sterol-sensing domain (SSD), intertwine tightly to form a stable domain (Kober et al., 2021; Yan et al., 2021a; Yan et al., 2021b). The domain may interact with the membrane to sense cholesterol (Kober et al., 2021). When cholesterol levels exceed 5% of the molar content of membrane lipids in the endoplasmic reticulum (ER), the interaction between SCAP’s loop 1 and loop 7 dissociates, allowing SCAP to bind to cholesterol, inducing SCAP to bind INSIG. (Radhakrishnan et al., 2008). This interaction promotes the binding of SCAP to INSIG, an ER-retention membrane protein, which inhibits the association of the SCAP-SREBP2 complex with COPII vesicles (Sun et al., 2005). Consequently, this effectively prevents the exit of the SCAP-SREBP2 complex from the ER. Additionally, Cholesterol not only promotes the binding of SCAP to INSIG proteins but also stabilizes INSIG, preventing its degradation via the ubiquitin-proteasome pathway (Gong et al., 2006; Lee et al., 2006). Conversely, when cholesterol levels decrease, the first and seventh loops of SCAP interconnect, enabling the SCAP-SREBP2 complex to bind to COPII vesicles, which facilitates its translocation to the Golgi apparatus (Sun et al., 2005; Brown et al., 2018) (Figure 4). Moreover, the transport of the SREBP2 is also regulated by other molecules. Multimeric endoplasmic reticulum (ER) proteins known as ERLINs, large tumor suppressor kinase 2 (LATS2), along with two ubiquitin ligases, TRC8 and RNF145, negatively regulate the egress of SREBP2 from the ER. ERLINs tightly interact with the SCAP-SREBP2-INSIG complex, while TRC8 directly binds to SREBP2 and SCAP (Irisawa et al., 2009; Huber et al., 2013). RNF145 ubiquitylates SCAP within a critical loop essential for COPII binding (Zhang L. et al., 2017). These events impair COPII binding and hinder the transport of SREBP2. LATS2 directly interacts with SREBP2 to retain it in the ER. However, MiR-96 reduced the levels of the SREBP2 anchor protein INSIG2 (Aylon et al., 2016). Phosphoenolpyruvate carboxykinase 1 (PCK1) can phosphorylate the Ser207 of INSIG1 and the Ser151 of INSIG2, thereby disrupting the interaction between INSIG proteins and SCAP. These result in an increase in transport of SREBP2(Xu et al., 2020). However, another study found that SREBP2 processing unperturbed by phosphorylated INSIG2, which exhibits exclusive inhibitory specificity toward SREBP1(Tian et al., 2024).

### 3.5 SREBP2 proteolytic activation in the Golgi

The SCAP–SREBP2 complex binds to COPII and is transported from the endoplasmic reticulum (ER) to the Golgi apparatus. Within the Golgi, the PAQR3 protein interacts with the SCAP–SREBP2 complex, retaining it in this organelle. Under cholesterol-depleting conditions, the transcription of the PAQR3 gene is upregulated, facilitating the retention process (Xu et al., 2015). SREBP2 undergoes proteolytic activation in a two-step process. The first step involves the cleavage of the luminal loop of SREBP2 by site 1 protease (S1P), producing a cleaved form of SREBP2 that is approximately half the size of the original protein; this cleavage is crucial for the subsequent activation step (Sakai et al., 1998). SPRING acts as an activating cofactor for S1P to involve in the proteolytic cleavage of SREBP2. Multiple studies have established that SPRING (previously C12ORF29) deficiency impairs SREBP2 processing (Bayraktar et al., 2020; Loregger et al., 2020). Current works demonstrate that SPRING facilitates S1P maturation to regulate SREBP2 activation (Bayraktar et al., 2020; Xiao et al., 2021; Hendrix et al., 2024a). Crucially, SPRING specifically enables a specific pool of S1P to execute SREBPs proteolytic cleavage, rather than being universally involved in all S1P-dependent pathways (Hendrix et al., 2024b). Additionally, SPRING has been reported to regulate the transport of SCAP (Aregger et al., 2020; Loregger et al., 2020). Following cleavage of SREBP2 by S1P, the N-terminal region of the SREBP2 protein undergoes a second proteolytic processing by site 2 protease (S2P) (Rawson et al., 1997). These cleavage events release a soluble N-terminal transcription factor known as n-SREBP2, which translocates to the nucleus as a homodimer (Brown et al., 2018). In the nucleus, n-SREBP2 binds to SRE sequences, thereby activating the transcription of target genes involved in cholesterol metabolism (Goldstein et al., 2006) (Figure 4).

### 3.6 Regulation of n-SREBP2 protein

The level and transcriptional activity of n-SREBP2 protein are regulated by various pathways. The mechanistic target of rapamycin complex 1 (mTORC1) upregulates n-SREBP2 protein level through two primary mechanisms. mTORC1 increases n-SREBP2 proteins by decreasing the nuclear entry of lipin 1, a phosphatidic acid phosphatase that downregulates the protein levels of n-SREBP2 in the nucleus (Peterson et al., 2011). mTORC1 can also activate SREBP2 by inhibiting cholesterol trafficking from lysosomes to the ER, which increases n-SREBP2 proteins level (Eid et al., 2017). Conversely, carbohydrate response element-binding protein (ChREBP) enhances the ubiquitination and proteasomal degradation of n-SREBP2, although the underlying mechanism remains unclear (Zhang D. et al., 2017). Phosphorylation of n-SREBP2 by serine/threonine protein kinase GSK3 targets it for proteasomal degradation via the SCF-FBW7 ubiquitin ligase complex, resulting in a reduction in n-SREBP2 levels (Sundqvist et al., 2005). However, miR-182 binds to the 3′UTR of FBW7, leading to a decrease in FBXW7 mRNA levels and a reduction in the degradation of n-SREBP2 protein (Jeon et al., 2013). Notably, the deubiquitinating enzyme USP28 has been shown to stabilize n-SREBP2 by reversing ubiquitination (Maier et al., 2023).

**FIGURE 5:**
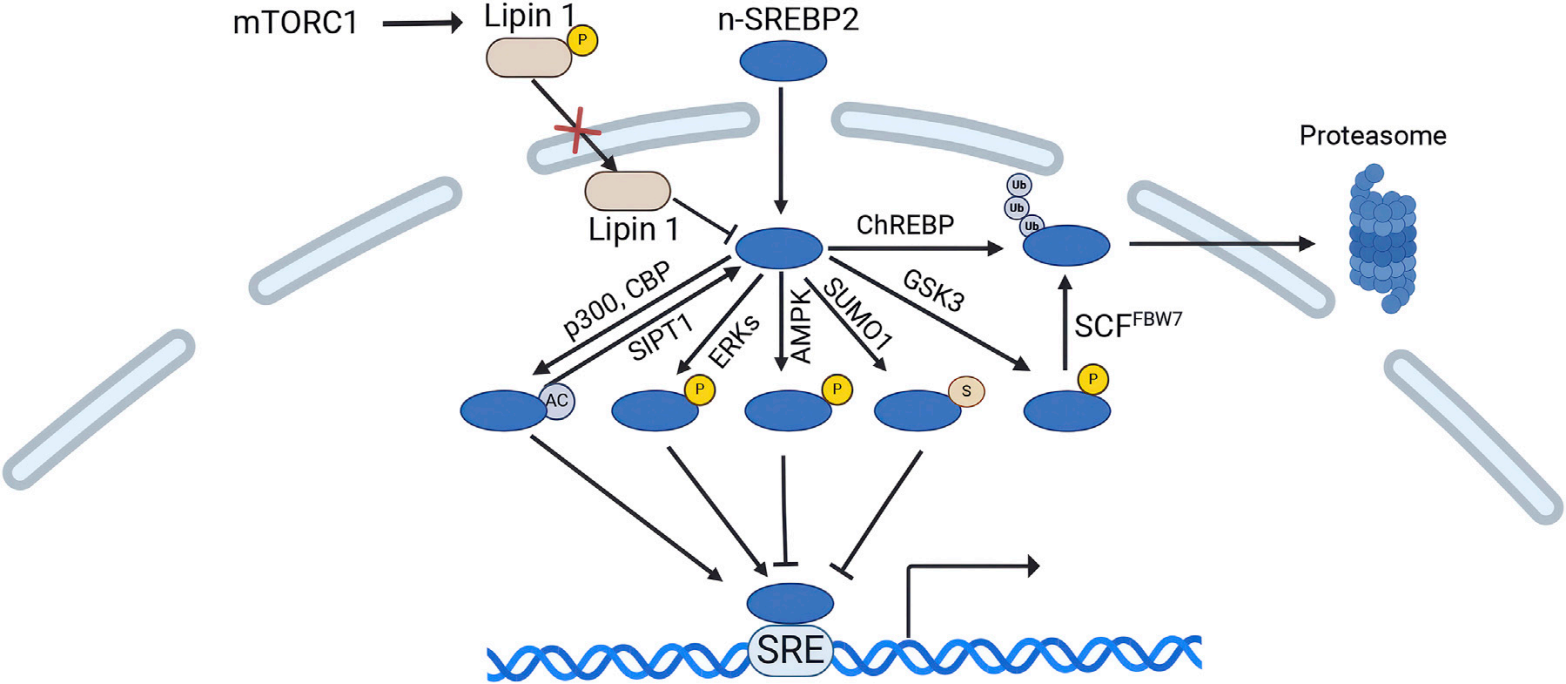
Regulation of n-SREBP2 protein. (Created in BioRender. Chen, R. (2024) https://BioRender.com/z88u544). mTORC1 phosphorylates lipin 1, reducing its nuclear entry. Lipin 1 decreases n-SREBP2 levels. The histone acetyltransferase p300 and its related protein CBP acetylate n-SREBP2 to enhance its transcriptional activity, while Sirtuin1 (SIRT1) deacetylates n-SREBP2. Additionally, phosphorylation of n-SREBP2 by ERK proteins also increases its transcriptional activity. Conversely, AMP-activated protein kinase (AMPK) phosphorylates n-SREBP2, inhibiting its nuclear translocation and transcriptional activity. SUMO1-mediated SUMOylation of n-SREBP2 also suppresses its transcriptional activity. Serine/threonine protein kinase GSK3 phosphorylates n-SREBP2, mediating its proteasomal degradation via the SCF-FBW7 ubiquitin ligase complex, thereby reducing n-SREBP2 levels. Carbohydrate response element-binding protein (ChREBP) also promotes n-SREBP2 ubiquitination and proteasomal degradation.

Importantly, the n-SREBP2 undergoes various post-translational modifications, including acetylation, phosphorylation, and sumoylation, which modulates the transcriptional activity of n-SREBP2. The histone acetyltransferase p300 and its related protein CBP can acetylate n-SREBP2 and increased its transcriptional activity, whereas deacetylation of Sirtuin1 (SIRT1) can deacetylate n-SREBP2 and counteract the effects of p300 and CBP(Giandomenico et al., 2003; Walker et al., 2010). SREBP2 has also been identified as a phosphorylation target of extracellular signal-regulated kinase (Erk) at Ser-455 and Ser-432, with Erk1/2-dependent phosphorylation upregulating SREBP2’s N-terminal transactivation (Bennett and Osborne, 2000; Kotzka et al., 2004). Conversely, AMP-activated protein kinase (AMPK) interacts with and directly phosphorylates n-SREBP2, inhibiting its nuclear translocation and transcriptional activity (Meng et al., 2024). SUMO1-mediated sumoylation at the sumoylation site of SREBP2 (Lys 464) also inhibits the transcriptional activity of n-SREBP2(Hirano et al., 2003) (Figure 5).

## 4 SREBP2 in cancer

Cholesterol homeostasis is crucial for both cellular and systemic functions. In cancer cells, cholesterol levels are significantly elevated to support rapid proliferation, resulting in increased uptake and storage of cholesterol within malignant tumors. As a key transcription factor regulating the expression of genes involved in cholesterol synthesis and uptake, SREBP2 connects oncogenic signaling with alterations in cholesterol metabolism, thereby playing a vital role in cancer development.

### 4.1 Liver cancer

There is a mounting body of evidence suggesting that SREBP2-mediated cholesterol biosynthesis plays a pivotal role in hepatocellular carcinoma (HCC) tumorigenesis (Calvisi et al., 2011; Che et al., 2020; Chen W. et al., 2022; Saito et al., 2023; Wang et al., 2023). In HCC, SREBP2 upregulates TAZ expression and increases TAZ interaction with TEAD2 by mediating cholesterol metabolism, thereby promotes HCC through enhanced DNA damage and associated proliferation (Saito et al., 2023). Furthermore, in the context of gut microbiota dysregulation, the increased levels of SREBP2 and heightened expression of cholesterol synthesis-related genes are associated with the reduction of tryptophan metabolites and weakened AhR activation, contributing to the initiation of liver cancer (Chen W. et al., 2022). Conversely, impaired SREBP2 maturation and suppressed cholesterol biosynthesis inhibit HCC cell proliferation, which is associated with a favorable prognosis in HCC patients (Feng et al., 2017; Liang et al., 2019; Xiang et al., 2022; He et al., 2024). In addition, SREBP2 plays a significant role in promoting the metastasis of liver cancer by enhancing the expression of genes involved in cellular migration and invasion. Nuclear translocation of SREBP2 is promoted by inhibition of LATS, significantly reducing E-cadherin expression while upregulating N-cadherin, Snail, and Vimentin, thereby facilitating HCC cell migration and invasion (Zhang et al., 2023). Beyond its role in promoting the progression of HCC, SREBP2 also significantly contributes to drug resistance in this malignancy. Cleavage of SREBP2 from the ER by Caspase-3 (CASP3) activates cholesterol biosynthesis, subsequently activating the sonic hedgehog signaling pathway, which renders cancer stem cell populations in HCC resistant to sorafenib and lenvatinib treatment (Mok et al., 2022). The study highlighting the need for therapeutic strategies that can effectively target SREBP2 to overcome resistance and improve patient responses to treatment. In conclusion, these studies suggest that SREBP2 participates in hepatocellular carcinogenesis and progression and targeting SREBP2 may represent a promising therapeutic strategy for liver cancer.

### 4.2 Pancreatic cancer

In pancreatic cancer, a growing body of research indicates that the activation of SREBP2 leads to alterations in cholesterol metabolism, thereby enhancing cancer cell resistance to apoptosis and promoting tumorigenesis and progression. KRAS mutations are prevalent in promoting pancreatic ductal adenocarcinoma (PDAC) (Duan et al., 2024). A study employing scRNA-seq revealed that the cholesterol synthesis pathway, including SREBP2, is specifically upregulated in KRAS mutant pancreatic organoids. These findings support a model in which oncogenic KRAS mutations activate SREBP2, leading to the reprogramming of cholesterol metabolism (Duan et al., 2024). The resultant cholesterol accumulation provides energy for pancreatic cancer growth and enhances resistance to apoptosis. Reducing the expression levels of SREBP2 can reverse the reprogramming of cholesterol metabolism induced by oncogenic KRAS (Zhang D. et al., 2021). Furthermore, SREBP2 interacts with transcription factor CP2 (TFCP2) in pancreatic cancer cells, allowing them to overcome KRAS mutation-induced senescence (Zhang D. et al., 2021). This interaction results in enhanced growth and metastasis of pancreatic cancer cells (Zhang D. et al., 2021). SREBP2 also forms a complex with β-catenin on the promoters of MVA pathway genes following the disruption of ciliogenesis, activating the transcription of cholesterol metabolism-related genes and further promoting PDAC development (Deng et al., 2018). Together, these findings underscore the significant role of SREBP2 in the tumorigenesis and progression of pancreatic cancer.

### 4.3 Colorectal cancer

An increasing body of evidence reveals that SREBP2-mediated cholesterol biosynthesis is involved in colorectal carcinogenesis, progression and change in energy homeostasis. Clinical studies have demonstrated that SREBP2 levels are elevated in early-stage colorectal cancer but diminished in late-stage disease (Sharma et al., 2019). SREBP2-mediated cholesterol metabolism participates in oncogenic pathways that drive and promote colorectal cancer (CRC). Dysregulated activity of Yes-associated protein (YAP) and mechanistic target of rapamycin complex 1 (mTORC1) is associated with tumorigenesis and progression (Pan et al., 2021). SREBP2 interacts with ZMYND8, which is upregulated by YAP, leading to increased cholesterol levels that subsequently activate mTORC1 and drive the colorectal carcinogenesis. This interaction highlights the SREBP2-mediated YAP/ZMYND8/mTORC1 pathway, which endows CRC cells with specific metabolic vulnerabilities (Pan et al., 2021). Similarly, in aggressive mesenchymal CRC, enhanced stabilization of SCAP and activation of SREBP2 also creates a cholesterol metabolic addiction and a therapeutic vulnerability (Muta et al., 2023). It makes statin-based inhibitors strongly suppress tumor growth (Muta et al., 2023). This heightened expression of SREBP2 plays a crucial role in the metabolic adaptations of CRC, highlighting the significance of cholesterol metabolism in this malignancy (Wen et al., 2018; Jin et al., 2023). In addition, SREBP2 also promotes the colorectal cancer metastasis. SREBP2 and SREBP2-dependent cholesterol biosynthesis are activated by c-Met/PI3K/AKT/mTOR axis in CRC, which allows CRC cells to undergo dynamic metabolic adjustments, facilitating adaptation to metastatic conditions (Zhang K. L. et al., 2021). This activation is required for the CRC cells metastasis, colonization and growth (Zhang K. L. et al., 2021). Collectively, these findings clarify that SREBP2 play a vital role in colorectal cancer development. Strategies targeting SREBP2-mediated cholesterol metabolism beneficial for the development of effective therapeutic for CRC.

### 4.4 Lung cancer

In lung cancer, downregulation of SREBP2 significantly inhibits the proliferation, migration, and invasion of cancer cells (Zhu et al., 2024). In addition, SREBP2 also promotes the occurrence of complication associated with lung cancer treatment. Lung cancer-related pleural effusion (LCPF), a common complication of lung cancer treatment, stimulates expression of SREBP2, which can induce pleural angiogenesis in patients and exacerbate advanced on-small cell lung cancer (NSCLC) with pleural effusion (Tsai et al., 2022). At present, drug resistance brings great challenges to the advanced treatment and prognosis of cancer patients. Activating the cleavage of SREBP2 increased the synthesis of cholesterol, leading to NSCLC cells more resistant to cisplatin (Akman et al., 2024). Another study found that SREBP2 and the cholesterol metabolism was highly activated in NSCLC cells resistance to osimertinib (Cao et al., 2024). Inhibiting SREBP2 is helpful for reversing NSCLC cells osimertinib acquired resistance (Cao et al., 2024). In summary, these results illustrate that SREBP2 participates in lung cancer progression, treatment complications, and drug resistance, and SREBP2 is a potential target for lung cancer treatment.

### 4.5 Breast cancer

In breast cancer, SREBP2 expression is upregulated, and its elevated levels contribute to tumor progression (Ricoult et al., 2016; Huang et al., 2017; Cai et al., 2019; Chen Y. Y. et al., 2022; Hunt et al., 2023). The transcription of SREBP2 is enhanced through phosphorylation of STAT3 (Tyr705) in triple-negative breast cancer (TNBC) (Chen Y. Y. et al., 2022). Additionally, RORγ mediates the chromatin recruitment and activation of SREBP2 in TNBC cells (Cai et al., 2019). Phosphorylated β-catenin also stimulates SREBP2 expression, promoting circulating tumor cell colony formation and tumor recurrence (Hunt et al., 2023). In contrast, inhibition of SREBP2 protein levels suppresses breast cancer cell proliferation. The cholesterol transporter ABCA9, which accumulates cholesterol in the endoplasmic reticulum (ER), reduces SREBP2 expression, thereby impairing breast cancer cell proliferation (Hwang et al., 2023). Furthermore, elevated SREBP2 expression is induced by CREB signaling, which subsequently upregulates NFATc1 expression required for mature osteoclast formation, contributing critically to breast cancer invasion and bone metastasis (Jie et al., 2019). Another study demonstrated that increased SREBP2 levels, mediated by the mTORC1 pathway, promote metastasis and a more malignant phenotype in breast cancer (Ning et al., 2023). Collectively, these findings indicate that the upregulation of SREBP2 drives tumorigenesis and progression in breast cancer.

### 4.6 Ovarian cancer

An expanding array of studies demonstrates that SREBP2 expression is increased and that cholesterol synthesis is facilitated in ovarian cancer (OC), which plays a pivotal role in tumorigenesis (Zhao et al., 2020). Decreasing SREBP2 levels can prevent statin-induced sterol feedback, thereby enhancing statin toxicity and efficacy in ovarian cancer cells (Casella et al., 2014). In addition to promoting tumor progression by upregulating cholesterol metabolism, SREBP2 can also promote the proliferation, migration, and epithelial-to-mesenchymal transition (EMT) of OC cells by directly activating the PRSS8/SCNN1A axis (Cai et al., 2021). Moreover, SREBP2 is implicated in mechanisms of drug resistance in OC. One study found that SREBP2 and its downstream genes were upregulated in cisplatin-resistant cells, indicating its involvement in mediating resistance to cisplatin (Zheng et al., 2018). Notably, blocking the SREBP2 pathway has been shown to increase cisplatin sensitivity in OC (Zheng et al., 2018). As such, these findings suggest that targeting SREBP2 and its related pathways may offer a promising strategy for developing effective therapeutic interventions for OC.

### 4.7 Endometrial cancer

Research generally indicates that inhibition of SREBP2 expression contributes to the suppression of endometrial cancer progression (Gao et al., 2018; Wang et al., 2021). For instance, the downregulation of SREBP2 by BF175, resulting in reduced cholesterol levels in endometrial cancer, enhance anti-tumor effect (Wang et al., 2021). Similarly, fatostatin inhibits the development of endometrial carcinoma by downregulating SREBP2 and interfering with SREBP2-mediated cholesterol metabolic pathways, further demonstrating anti-tumor effect of downregulated SREBP2 (Gao et al., 2018). Contrastingly, another study reported low expression levels of SREBP2 and AMPK in endometrial cancer tissues, noting that SREBP2 is a target gene inhibited by AMPK (Efsun Antmen et al., 2021). This discrepancy may be attributed to variations in the samples studied and the context of SREBP2 regulation in different tumor microenvironments. Overall, these findings highlight the complex role of SREBP2 in endometrial cancer, suggesting that its regulation may have therapeutic implications.

### 4.8 Prostate cancer

Androgens play a critical role in maintaining the survival and proliferation of prostate cancer (PCa) by binding to and activating the androgen receptor (AR) (Fujita and Nonomura, 2019). Consequently, lipid synthesis and uptake are vital energy resources that support tumor progression in PCa. A study comparing prostate cancer tissue with benign prostate tissue through single-cell sequencing revealed heightened activity in cholesterol metabolism and underscored the essential role of SREBP2 in prostate cancer progression (Wei et al., 2024). In castration-resistant prostate cancer (CRPC), the SREBP2 is activated by PTEN/p53 deficiency, thereby upregulating cholesterol metabolism and facilitating tumor cell survival and growth (Shangguan et al., 2022). Another study also found that increasing SREBP2 transcription contributes to the malignant characteristics of prostate adenocarcinoma (Lin et al., 2022). Conversely, downregulated of SREBP2 in prostate cancer cells leads to suppressed cancer progression (Li et al., 2013; Li et al., 2014; Longo et al., 2019). Additionally, studies have shown that SREBP2 and its downstream effector genes are upregulated in prostate cancer following androgen ablation, triggering the formation of androgen-independent (AI) tumors (Ettinger et al., 2004). Targeting SREBP2 effectively inhibited tumor growth and metastasis (Wei et al., 2024). Collectively, these findings suggest that inhibiting SREBP2 represents a promising therapeutic strategy for prostate cancer.

### 4.9 Bladder cancer

In bladder cancer, a study indicates that SREBP2 interacts with CBP and NFYC-37, activating the transcription of genes involved in the mevalonate pathway, thereby promoting cholesterol biosynthesis and tumor growth (Liu et al., 2023). In studies utilizing mRNA/miRNA microarrays and protein analysis in T24 bladder cancer cells, archazolid B was found to activate SREBP2, resulting in severely deregulated cholesterol homeostasis and contributing to archazolid B-induced resistance (Hamm et al., 2014). Additionally, SREBP2 plays an important in metastasis of bladder cancer. Decreasing SREBP2 expression by the Farnesoid X Receptor (FXR) has been shown to suppress lung metastasis in bladder cancer (Lai et al., 2022). Combining with cholesterol suppression treatment can further inhibits the migratory, invasive, and angiogenic properties of human urothelial carcinoma, including bladder cancer (Lai et al., 2022). These results suggest that targeting SREPB2 seems to have potential as a strategy for bladder cancer treatment.

### 4.10 Glioblastoma

In glioblastoma (GBM), SREBP2 is highly expressed and plays a crucial role in upregulating cholesterol biosynthesis in glioblastoma stem-like cells (GSCs), promoting tumor proliferation, self-renewal, and overall tumor growth (Gu et al., 2023). Retention of cholesterol impairs its intracellular delivery in GSCs, subsequently triggering the SREBP2 transcriptional program to meet cholesterol demands (Maghe et al., 2024). Conversely, reduced SREBP2 expression can exacerbate autophagy defects and increase cell death in GSCs, consequently suppressing tumor progression (Maghe et al., 2024). Additionally, oxygen and nutrient limitations in the tumor microenvironment are closely associated with cancer progression (Lewis et al., 2015). In glioblastoma multiforme, inhibiting SREBP2 function has been shown to block lipid biosynthesis in hypoxic cancer cells, impairing their survival under hypoxic conditions (Lewis et al., 2015). These results illustrate that SREBP2-mediated cholesterol metabolism participates in glioblastoma progression. Furthermore, decreasing the expression of the SREBP2 gene in glioblastoma can suppress mesenchymal transformation in non-mesenchymal gliomas, thereby inhibiting tumor metastasis (Ferrarese et al., 2023). Furthermore, lipid synthesis pathways mediated by SREBP2 are implicated in the resistance mechanism to temozolomide (TMZ), a common chemotherapy for GBM (Choo et al., 2023). Inhibiting SREBP2 enhances sensitivity to TMZ therapy, indicating its potential as a therapeutic target (Choo et al., 2023).

### 4.11 Melanoma

In melanoma, the SREBP2 pathway is activated by different ways, leading to high levels of cholesterol biosynthesis, which in turn sustains the rapid proliferation of melanoma cells both *in vivo* and *in vitro* (Yamauchi et al., 2011; Dinavahi et al., 2022). Additionally, in addition to promoting tumor progression by upregulating cholesterol metabolism, SREBP2 can also reduce reactive oxygen species (ROS) and lipid peroxidation, which confers resistance to inducers of ferroptosis in melanoma cells, thereby facilitating tumor progression (Hong et al., 2021).

### 4.12 Other cancers

In gastric cancer, expression of SREBP2 and cholesterol synthesis are promoted by sterol O-acyltransferase 1 (SOAT1), which facilitates cancer cells lymph node metastasis (Zhu et al., 2021). Another study shows that gastric cancer stem cells enhance the expression of SREBP2 to augment cholesterol metabolism, thereby altering tumor cell membranes and increasing their resistance to perforin released by natural killer (NK) cells (Zhu and Wang, 2024). In esophageal squamous cell carcinoma (ESCC), SREBP2 interacts with c-Myc, synergistically inducing the expression of HMGCR, which promotes ESCC migration, invasiveness, viability, and anchorage-independent growth (Zhong et al., 2019). Additionally, lysophosphatidylcholine acyltransferase 1 (LPCAT1) regulates the nuclear translocation of SREBP2, thereby promoting the proliferation of ESCC cells (Tao et al., 2021). In clear cell renal cell carcinoma (ccRCC), SREBP2 interacts with MED15 that acts as a SREBP2 coactivator to promote cholesterol biosynthesis enzyme expression, resulting in enhancing malignant tumor behavior phenotypes (Hua et al., 2024). PI3K/AKT/mTOR/SREBP2 pathway is upregulated by VHL mutations and the subsequent stabilization of HIFα, contributing to the accumulation of intracellular cholesteryl esters and facilitating ccRCC development (Zhang et al., 2024). In osteosarcoma, research indicates that SREBP2 phosphorylation at T610 by PKM2 enhances its stability and promoting tumorigenesis (Pu et al., 2022). In t (4; 11) leukemia, SREBP2 is significantly overexpressed and correlates with a poorer prognosis (Erkner et al., 2024). In summary, SREBP2 is significantly involved in the occurrence and development of various tumors (Table 1).

**TABLE 1:** Molecular mechanism of SREBP2 in cancer from the finding.

Cancer type	Functional Impact	Molecular mechanism from the findings	References
Liver cancer	Proliferation, DNA damage	SREBP2-mediated cholesterol metabolism upregulates TAZ expression and increases TAZ interaction with TEAD2 to enhance DNA damage and proliferation	Saito et al. (2023)
	tumorigenesis	SREBP2-mediated cholesterol metabolism contributes to the initiation of liver cancer in the context of gut microbiota dysregulation	Chen et al. (2022a)
	Metastasis	LATS upregulates SREBP2 to suppress E-cadherin and induce N-cadherin/Snail/Vimentin, thereby promoting HCC metastasis	Zhang et al. (2023)
	Drug resistance	Caspase-3 activates SREBP2-mediated cholesterol metabolism, which sequentially induces SHH signaling, driving chemoresistance in HCC cancer stem cells	Mok et al. (2022)
Pancreatic cancer	Apoptosis	KRAS mutation activates SREBP2, driving cholesterol reprogramming to enhance apoptosis resistance in PDAC.	Duan et al. (2024)
	Metastasis	The interaction between SREBP2 and TFCP2 promotes PDAC progression and metastasis	Zhang et al. (2021a)
	Tumorigenesis	SREBP2 forms a complex with β-catenin to activate transcription of cholesterol metabolism genes, thereby promoting PDAC progression	Deng et al. (2018)
Colorectal cancer	Tumorigenesis	SREBP2-mediated YAP/ZMYND8/mTORC1 pathway drives CRC development	Pan et al. (2021)
	Therapeutic vulnerability	Activation of SREBP2-mediated cholesterol metabolism creates a cholesterol a therapeutic vulnerability in aggressive mesenchymal CRC.	Muta et al. (2023)
	Metastasis	The c-Met/PI3K/AKT/mTOR signaling axis activates SREBP2-Mediated cholesterol metabolism to promote CRC adaptation to metastatic condition	Zhang et al. (2021b)
Lung cancer	Pleural angiogenesis	LCPF stimulates SREBP2 expression to induce pleural angiogenesis	Tsai et al. (2022)
	Drug resistance	Activation of SREBP2-mediated cholesterol metabolism confers enhanced cisplatin and osimertinib resistance in NSCLC.	Akman et al. (2024), Cao et al. (2024)
Breast cancer	Growth	STAT3 phosphorylation (Tyr705) enhances SREBP2 transcription to regulate TNBC cell growth	Chen et al. (2022b)
	Metabolic reprogramming	RORγ mediates the chromatin recruitment and activation of SREBP2 in TNBC.	Cai et al. (2019)
	Proliferation	Phosphorylated β-catenin stimulates SREBP2 expression, promoting circulating tumor cell colony formation and tumor recurrence in breast cancer	Hunt et al. (2023)
	Proliferation	ABCA9 accumulates cholesterol in ER, reduces SREBP2 expression, thereby impairing breast cancer cells proliferation	Hwang et al. (2023)
	Invasion, Metastasis	CREB induces SREBP2-driven NFATc1 upregulation, promoting breast cancer invasion and bone metastasis	Jie et al. (2019)
Ovarian cancer	Statin toxicity	Decreasing SREBP2 levels prevent statin-induced sterol feedback, thereby enhancing statin toxicity and efficacy in OC cells	Casella et al. (2014)
	Proliferation, Migration, EMT	SREBP2 activates the PRSS8/SCNN1A axis to promote OC proliferation, migration, and EMT.	Cai et al. (2021)
	Drug resistance	Upregulated SREBP2 mediates resistance to cisplatin in OC.	Zheng et al. (2018)
Endometrial cancer	Cancer progression	Pharmacological inhibition of SREBP2 by BF175 and fatostatin impedes endometrial cancer progression	Wang et al. (2021), Gao et al. (2018)
Prostate cancer	Proliferation	SREBP2-mediated cholesterol metabolism is activated by PTEN/p53 deficiency, thereby facilitating prostate cancer cell survival and growth	Shangguan et al. (2022)
	Anti-tumor activity	The upregulated SREBP2 in prostate cancer following androgen ablation triggers the development of CRPC.	Ettinger et al. (2004)
	Proliferation, Metastasis	Targeting SREBP2 effectively inhibited prostate cancer growth and metastasis	Wei et al. (2024)
Bladder cancer	Proliferation	SREBP2 interacts with CBP and NFYC-37, upregulating cholesterol biosynthesis, thereby promoting cholesterol biosynthesis and bladder cancer growth	Liu et al. (2023)
	Metastasis	FXR upregulates SREBP2 expression to promote lung metastasis in bladder cancer	Lai et al. (2022)
Glioblastoma	Proliferation, Self-renewal	SREBP2 upregulates cholesterol biosynthesis, promoting GSCs proliferation, self-renewal, and overall tumor growth	Gu et al. (2023)
	Autophagy	Reduced SREBP2 expression exacerbates autophagy defects and increase cell death in GSCs	Maghe et al. (2024)
	Survival	Inhibiting SREBP2 function show to block lipid biosynthesis in hypoxic cancer cells, impairing their survival under hypoxic conditions	Lewis et al. (2015)
	Metastasis	Decreasing the expression of the SREBP2 suppresses mesenchymal transformation in non-mesenchymal gliomas, thereby inhibiting tumor metastasis	Ferrarese et al. (2023)
	Drug resistance	Activation of SREBP2-mediated cholesterol metabolism mediates resistance to TMZ in Melanoma	Choo et al. (2023)
Melanoma	Proliferation	SREBP2-mediated high level of cholesterol biosynthesis sustains the rapid proliferation of melanoma	Yamauchi et al. (2011), Dinavahi et al. (2022)
	Ferroptosis	SREBP2 reduces ROS and lipid peroxidation, conferring resistance to ferroptosis in melanoma	Hong et al. (2021)
Gastric cancer	Metastasis	SREBP2-mediated cholesterol metabolism is activated by SOAT1, thereby facilitating cancer cells lymph node metastasis	Zhu et al. (2021)
	Resistance to NK cell cytotoxicity	Increased expression of SREBP2 increases the resistance of gastric cancer stem cells to perforin released by natural killer cells	Zhu and Wang (2024)
Esophageal squamous cell carcinoma	Migration, Invasiveness	SREBP2 interacts with c-Myc, promoting ESCC migration, invasiveness, viability, and anchorage-independent growth	Zhong et al. (2019)
	Proliferation	LPCAT1 regulates the nuclear translocation of SREBP2, promoting the proliferation of ESCC cell	Tao et al. (2021)
Clear cell renal cell carcinoma	Proliferation, Migration, Invasion	SREBP2 interacts with MED15, resulting in enhancing ccRCC malignant tumor behavior phenotypes	Hua et al. (2024)
	Proliferation, Migration, Invasion	PI3K/AKT/mTOR/SREBP2 pathway is upregulated by VHL mutations and the subsequent stabilization of HIFα, facilitating ccRCC development	Zhang et al. (2024)
Osteosarcoma	Tumorigenesis	SREBP2 phosphorylation at T610 by PKM2 enhances osteosarcoma stability and promoting tumorigenesis	Pu et al. (2022)

## 5 SREBP2 in tumor microenvironment

As previously discussed, SREBP2 signaling pathway is frequently activated in cancer cells, leading to increased cholesterol biosynthesis and uptake that promotes proliferation. However, the tumor microenvironment (TME) is a critical component of cancer, and current understanding of tumorigenesis and progression is increasingly shifting from a tumor cell-centric view to one that embraces the complexity of the tumor ecosystem (de Visser and Joyce, 2023). Consequently, recent many researches have focused on the regulation of SREBP2 within the TME. CD8^+^ T cells, which are immune cells capable of recognizing and eliminating cancer cells, are exposed to oxidized sterols secreted by cancer cells into the tumor microenvironment (TME) (Yan et al., 2023). These oxidized sterols significantly suppress SREBP2 activity in CD8^+^ T cells, resulting in cholesterol depletion and autophagy-mediated T cell apoptosis or dysfunction (Yan et al., 2023). Another study employing fatostatin to inhibit SREBP2 found that reduced cholesterol levels in the TME led to a decreased proportion of regulatory T (Treg) cells and alleviated CD8^+^ T cell exhaustion, thereby enhancing antitumor activity (Zhu et al., 2024). However, recent studies have shown that the upregulation of SREBP2 also supports CD8^+^ T cell function, not through cholesterol metabolism, but by mediating the synthesis of non-steroidal products such as coenzyme Q (CoQ) (Reina-Campos et al., 2023). This process promotes their differentiation into tissue-resident memory CD8^+^ T (TRM) cells, thereby enhancing antitumor immunity (Reina-Campos et al., 2023). Natural killer T (NKT) cells can mediate immune responses in cancer. Obesity-induced activation of SREBP2 and altered cholesterol metabolism in liver lead to hypercholesterolemia, resulting in excessive cholesterol uptake by NKT cells (Tang et al., 2022). This leads to the accumulation of lipid peroxides in NKT cells, thereby impairing their antitumor immunosurveillance capabilities (Tang et al., 2022). Furthermore, in melanoma, it has been observed that cancer-derived lactate activates SREBP2 in conventional dendritic cells (DCs), driving their transformation into regulatory DCs (mregDCs) (Plebanek et al., 2024). These mature mregDCs suppress DC antigen cross-presentation, thereby promoting melanoma progression (Plebanek et al., 2024).

## 6 Targeting SREBP2 for cancer therapy

As outlined above, abnormal expression and activation of SREBP2 play a significant role in the initiation and progression of various cancers. Consequently, targeting SREBP2 maturation or transcriptional activity has emerged as a high priority in cancer therapy. Recent studies have identified various SREBP2 inhibitors, and findings regarding several of these agents for cancer treatment are summarized below (Table 2).

**TABLE 2:** SREBP2 inhibitor.

SREBP2 inhibitor	Target	Cancer type	IC50	Dose and intervention time in animol model
Fatostatin	SCAP	Prostate cancer	0.1 µM (DU-145)	15 mg/kg for 42–60 days
			9.1 µM (C4-2B)	
			10.4 µM (LNCaP)	
		Endometrial carcinoma	17.96 µM (Ishikawa)	
			4.53 µM (HEC-1A)	
		Breast cancer	**-**	
		Glioblastoma multiforme	**-**	
		Acute lymphoblastic leukemia	**-**	
		Hepatocellular carcinoma	**-**	
Betulin	SCAP-INSIG	Prostate cancer	24 h	**-**
			4.921 μg/mL (LNCaP)	
			2.936 μg/mL (PC3)	
			48 h	
			7.347 μg/mL (LNCaP)	
			3.035 μg/mL (PC3)	
		Breast cancer	**-**	
		Hepatocellular carcinoma	**-**	
		Lung cancer	**-**	
Xanthohumol	SCAP-SREBP2 complex	Hepatocellular carcinoma	-	300 mg/kg for 56 days
PF-429242	S1P	Hepatocellular carcinoma	0.5 µM(HepG2)	30 mg/kg
		glioblastoma	full serum media	
			15 ± 1.3 mM (T98G)	
			lipoprotein-deficient	
			0.32 ± 0.09 mM (T98G)	
			15.2 ± 3.0 Mm (U87-MG)	
			27.6 ± 5.3 mM (A172)	
BF175	-	endometrial cancer		0.3 mg/g/week

### 6.1 Fatostatin

Fatostatin, a non-sterol diarylthiazole derivative, has been investigated for the treatment of multiple tumors. Originally developed from a chemical library in 2003 and referred to as 125B11, fatostatin has demonstrated promising antitumor effects (Choi et al., 2003). It inhibited IGF1-induced growth in prostate cancer DU-145 cells with an half-maximal inhibitory concentrations (IC50) of 0.1 µM (Choi et al., 2003). Mechanistically, fatostatin blocks the ER-Golgi translocation of SREBP2 by directly binding to SCAP at a site distinct from the sterol-binding domain, thereby inhibiting the cleavage and activation of SREBP2 (Kamisuki et al., 2009). In prostate cancer, fatostatin reduces the secretion of extracellular vesicles (EVs) from hypoxia-stimulated cancer cells by inhibiting cholesterol biosynthesis, which decreases tumor EMT, invasiveness, and stemness (Schlaepfer et al., 2015). A study utilizing human prostate cancer cell lines demonstrated that fatostatin inhibits cell proliferation, invasion, and migration while inducing caspase-mediated apoptosis, with IC50 (72 h of treatment) of 9.1 µM and 10.4 µM in C4-2B and LNCaP cells, respectively (Li et al., 2014). *In vivo*, fatostatin exhibited antitumor efficacy in a subcutaneous C4-2B xenograft mouse model when administered at a dose of 15 mg/kg for 42 days (Li et al., 2014). Another study also indicated that treatment with fatostatin (15 mg/kg) for 60 days in the Pmlpc^−/−^ mouse prostate tumor metastatic model effectively suppressed the SREBP pathway and increased antimetastatic activity (Chen et al., 2018). In androgen receptor-negative prostate cancer cells harboring mutant p53, fatostatin also inhibited cell growth and induced apoptosis (Li et al., 2015). Similar to its effects in prostate cancer, fatostatin has been shown to inhibit growth and proliferation while inducing caspase-mediated apoptosis in endometrial carcinoma, with IC50 (after 72 h of treatment) of 17.96 µM and 4.53 µM in Ishikawa and HEC-1A cells, respectively (Gao et al., 2018; Yao et al., 2020). In breast cancer, fatostatin prevents SREBP2 activation, attenuating osteoclastogenesis and breast cancer-induced osteolysis *in vivo*, thereby providing therapeutic benefits for patients with osteolytic bone lesions (Jie et al., 2019). In HCC, fatostatin markedly suppressed the EMT process (Zhang et al., 2023). Studies focusing on the tumor microenvironment have revealed that fatostatin decreases SREBP2 activation and intracellular cholesterol accumulation, leading to a reduction in regulatory T (Treg) cells—immunosuppressive cells—while alleviating CD8^+^ T cell exhaustion in the TME (Zhu et al., 2024). These findings confirm fatostatin’s promise in tumor immunotherapy by inhibiting SREBP2 activation. In acute lymphoblastic leukemia (ALL), fatostatin disrupts cholesterol metabolism, effectively counteracting drug tolerance by inducing cell death and repressing stemness (Malyukova et al., 2024). In glioblastoma multiforme, fatostatin has shown potential in overcoming temozolomide resistance (Choo et al., 2023). Despite its potential therapeutic value, fatostatin has been shown to inhibit the growth of control cells due to its toxic effects (Li et al., 2014; Erkner et al., 2024). Consequently, clinical trials involving fatostatin have not advanced, likely due to safety concerns associated with the compound. The *in vivo* activity and pharmacokinetics of fatostatin are both lower than expected (Peng et al., 2023). In addition, a study has found that fatostatin has additional pathway targets rather than specifically blocking SCAP’s ER-to-Golgi transport (Shao et al., 2016). Therefore, continued efforts should be made to develop specific SCAP inhibitors.

### 6.2 Betulin

Betulin is a pentacyclic triterpene abundantly found in birch bark (Alakurtti et al., 2006). Unlike fatostatin, which directly binds to SCAP to inhibit the translocation of SREBP2 to the Golgi, the tumor-suppressive effects of betulin primarily rely on enhancing the interaction between SCAP and INSIG (Tang et al., 2011, 2021). This interaction obstructs SCAP from binding to COPII vesicles, preventing the SCAP-SREBP2 complex from exiting the endoplasmic reticulum (ER) and thereby inhibiting SREBP2 activation (Tang et al., 2011, 2021). A study on prostate cancer demonstrated that betulin significantly downregulated SREBP2 and its target genes, exhibiting inhibitory effects on the downstream activity of the androgen receptor (AR), which suppressed prostate cancer progression (Wei et al., 2024). The IC50 values of betulin for LNCaP and PC3 cell lines at 24 h were 4.921 μg/mL and 2.936 μg/mL, respectively. At 48 h, the IC50 values for LNCaP and PC3 cells were 7.347 μg/mL and 3.035 μg/mL, respectively (Wei et al., 2024). In a study focusing on obesity-induced breast cancer, betulin was shown to inhibit SREBP2 processing in MCF-7 cells (McClellan et al., 2022). This reduction led to decreased survival and proliferation of breast cancer cells (McClellan et al., 2022). In HCC, treatment with betulin effectively alleviated inflammatory responses and HCC development *in vivo* by targeting the SREBP2 pathway (Li et al., 2017). In terms of tumor resistance, betulin enhanced gefitinib sensitivity in lung cancer both *in vitro* and *in vivo* (Li et al., 2016). Treatment with betulin and gefitinib significantly reduced tumor growth by inhibiting SREBP2 and targeting tumor metabolism (Li et al., 2016). Studies in rat and dog models indicated that betulin exhibited very low toxicity, with no severe adverse effects observed *in vitro* (Jäger et al., 2008). However, Betulin has poor water solubility; therefore, preclinical studies on more soluble forms of betulin are required (Adepoju et al., 2023).

### 6.3 Xanthohumol

Xanthohumol (XN) is a prenylated flavonoid derived from natural food constituents, recognized for its antioxidant and anti-inflammatory properties, which contribute to its antitumor effects (Stevens et al., 2000; Lupinacci et al., 2009). Shingo Miyata et al. demonstrated that XN acts as a novel inactivator of SREBP2. Mechanistically, Sec23/24 is a heterodimer recruited to the ER membrane, driving the formation of COPII vesicles (Espenshade et al., 2002). The binding of XN to Sec23/24 disrupts the incorporation of the SCAP/SREBP2 complex into COPII, preventing its ER-to-Golgi translocation and thus inhibiting SREBP2 activation (Espenshade et al., 2002; Miyata et al., 2015). *In vitro*, treatment with 10 µM XN significantly decreased the production of mature SREBP2 in human hepatoma Huh-7 cells (Miyata et al., 2015). *In vivo*, mature SREBP-2 levels were markedly downregulated in apolipoprotein E-deficient (ApoE^−/−^) mice fed a Western-type diet and treated with XN (300 mg/kg body weight/day) for 8 weeks (Doddapattar et al., 2013). However, current data are insufficient to systematically evaluate the anticancer activity of XN, highlighting the need for further *in vivo* studies to enhance understanding of its clinical efficacy (Harish et al., 2021). Additionally, XN also suffers from low oral bioavailability (Legette et al., 2014).

### 6.4 PF-429242

PF-429242 is an aminopyrrolidineamide that inhibits endogenous SREBP2 processing and decreases SREBP2 activation through pharmacological inhibition of S1P activity (Hawkins et al., 2008). PF-429242 inhibited GSK343-induced SREBP2 activation, contributing to its anticancer activity in HCC(Yang et al., 2019). Furthermore, research has demonstrated that PF-429242 suppresses the expression of SREBP2 target genes and cholesterol synthesis in hepatoma HepG2 cells, exhibiting an IC50 of 0.5 µM. In a vivo research indicated that treatment with PF-429242 at a dose of 30 mg/kg for 24 h, administered every 6 h, effectively reduced the expression of hepatic SREBP2 target genes and lowered cholesterol levels in mice (Hawkins et al., 2008). In a series of *in vitro* experiments, PF-429242 was identified as an anti-glioblastoma agent by suppressing SREBP2 activation, inhibiting cell growth and inducing apoptotic cell death (Caruana et al., 2017). The IC50 of PF-429242 in T98G cells with full serum media was 15 ± 1.3 mM for 72 h, which was higher than the IC50 of 0.32 ± 0.09 mM in lipoprotein-deficient serum, indicating that extracellular cholesterol reduces the potency of PF-429242 (Caruana et al., 2017). They also assessed the IC50 of PF-429242 in two other human glioblastoma cell lines, U87-MG and A172, which were found to be 15.2 ± 3.0 mM and 27.6 ± 5.3 mM, respectively (Caruana et al., 2017). However, PF-429242 presents certain medicinal limitations, including a high clearance rate and poor oral bioavailability (Hawkins et al., 2008).

### 6.5 BF175

Xiaoping Zhao et al. designed and synthesized several boron-containing bioactive stilbene derivatives, among which BF175 was identified as an inhibitor of SREBP2 expression and transcriptional activity (Zhao et al., 2014). In a high-fat diet mouse model, treatment with BF175 (0.3 mg/g body weight/week) for 1 week significantly decreased the mRNA levels of SREBP2 and cholesterol biosynthesis *in vivo* (Zhao et al., 2014). Unlike the direct inhibition of SREBP1a by blocking the MED15-KIX and SREBP-1a-TAD interaction, the mechanism by which BF175 suppresses SREBP2 expression and transcriptional activity remains unclear and requires further investigation (Zhao et al., 2014). In endometrial cancer, treatment of AN3CA cells with varying concentrations of BF175 for 24 h resulted in a significant, dose-dependent reduction in SREBP2 expression, demonstrating antitumor activity (Wang et al., 2021). Additionally, BF175 exhibited low toxicity in tissue culture, suggesting it may be an ideal candidate as an SREBP2 inhibitor for cancer treatment (Zhao et al., 2014).

## 7 Conclusion and future directions

This review has provided an overview of the structure and regulation of SREBP2, highlighting its functions and mechanisms in various cancers. As a central transcription factor in cholesterol metabolism, SREBP2 plays a crucial role in malignancy by linking oncogenic signaling pathways with alterations in lipid metabolism. Additionally, SREBP2 also exerts tumorigenic effects through several non-cholesterol-related pathways. Numerous signaling molecules regulate the transcription, activation, and stability of SREBP2, driving tumorigenesis and progression. Additionally, while only a limited number of studies have explored this aspect, SREBP2 also influence inflammation, immunity and tumor microenvironment—processes that are intricately connected to tumor biology. Future studies should focus on elucidating the relationship between SREBP2-mediated immunity and tumorigenesis, as well as cancer progression. A more comprehensive understanding of these interactions may offer valuable insights into the role of SREBP2 in cancer and uncover novel therapeutic strategies targeting antitumor immunity.

Given that SREBP2 is involved in the progression of various cancers, it holds promise as both a prognostic biomarker and a potential therapeutic target, making the development of SREBP2 inhibitors a key component of future cancer treatment strategies. While several SREBP2 inhibitors have shown anticancer effects in preclinical models, they have not yet entered clinical trials. The development of these inhibitors faces several challenges, including issues related to poor solubility, insufficient potency, and rapid metabolism, which hinder their progression into clinical use. Overcoming these hurdles will require the establishment of efficient high-throughput screening systems for SREBP2 inhibitors and the rational design of compounds with improved pharmacological properties. In addition, current inhibitors lack specificity for SREBP2. In the SREBP family, SREBP1 and SREBP2 have the same activation pathways, which leads to inhibitors targeting both proteins simultaneously. Furthermore, these agents may exert effects on other oncogenic pathways. Fatostatin, Betulin, and Xanthohumol have been shown to suppress cancer progression not only via the SCAP-SREBP pathway but also through inhibition of mTOR, MAPK, and Notch signaling (Kunnimalaiyaan et al., 2015; Han et al., 2019; Cai et al., 2023). These multi-target inhibitors still require more research regarding their efficacy and safety. SREBP2-interacting proteins or therapeutic antibodies may offer superior target selectivity, though these approaches remain unexplored to date. Recent studies identified UT-59 as a SCAP-specific inhibitor that could open the door to developing therapeutic leads for cancer; however, its therapeutic efficacy and mechanistic details require further experimental validation (Xu et al., 2024). Given the critical role of the basic helix-loop-helix (bHLH) domain in SREBP2 function, designing inhibitors targeting this region represents a promising therapeutic development strategy. In conclusion, SREBP2 is a key regulator of cholesterol metabolism and plays an integral role in cancer progression. Targeting SREBP2 directly may present a promising anticancer strategy. Further investigation into its regulatory mechanisms in tumors, coupled with the development of more effective inhibitors, holds the potential to offer new, impactful therapeutic opportunities for cancer treatment.
